# Prediction of antibody binding to SARS-CoV-2 RBDs

**DOI:** 10.1093/bioadv/vbac103

**Published:** 2023-01-02

**Authors:** Eric Wang

**Affiliations:** Institute for Medical Engineering and Science, Massachusetts Institute of Technology, 77 Massachusetts Avenue, Cambridge, MA 02139, USA

## Abstract

**Summary:**

The ability to predict antibody–antigen binding is essential for computational models of antibody affinity maturation and protein design. While most models aim to predict binding for arbitrary antigens and antibodies, the global impact of SARS-CoV-2 on public health and the availability of associated data suggest that a SARS-CoV-2-specific model would be highly beneficial. In this work, we present a neural network model, trained on ∼315 000 datapoints from deep mutational scanning experiments, that predicts escape fractions of SARS-CoV-2 RBDs binding to arbitrary antibodies. The antibody embeddings within the model constitute an effective sequence space, which correlates with the Hamming distance, suggesting that these embeddings may be useful for downstream tasks such as binding prediction. Indeed, the model achieves Spearman correlation coefficients of 0.46 and 0.52 on two held-out test sets. By comparison, correlation coefficients calculated using existing structure and sequence-based models do not exceed 0.28. The correlation coefficient against dissociation constants of antibodies binding to SARS-CoV-2 RBD variants is 0.46. Additionally, the residue-level escapes are highest in the antibody epitope, correlating well with experimentally measured escapes. We further study the effect of antibody chain use, embedding dimension size and feed-forward and convolutional architectures on the model results. Lastly, we find that the inference time of our model is significantly faster than previous models, suggesting that it could be a useful tool for the accurate and rapid prediction of antibodies binding to SARS-CoV-2 RBDs.

**Availability and implementation:**

The model and associated code are available for download at https://github.com/ericzwang/RBD_AB.

**Supplementary information:**

Supplementary data are available at *Bioinformatics Advances* online.

## 1 Introduction

The global impact of severe acute respiratory syndrome coronavirus 2 (SARS-CoV-2) and its variants is well known. In efforts to better understand the molecular mechanisms of the virus, particular focus has been given to the receptor-binding domain (RBD) because of its essential role in facilitating viral entry into host cells by binding to the receptor angiotensin-converting enzyme 2 (ACE2) (Hoffmann *et al.*, 2020; Zamorano Cuervo and Grandvaux, 2020). This is not to neglect other important regions of the viral spike protein (McCallum *et al.*, 2021; Örd *et al.*, 2020) or other viral proteins (Cubuk *et al.*, 2021; Sui *et al.*, 2021), but work focusing solely on the RBD has been very fruitful (Pino *et al.*, 2021; Starr *et al.*, 2020; Wang *et al.*, 2021; Wang and Chakraborty, 2022). Indeed, many mutations that emerge in notable variants reside in the RBD (Cai *et al.*, 2021) and many antibodies elicited by infection or vaccines target the RBD (Barnes *et al.*, 2020; Guo *et al.*, 2021; Muecksch *et al.*, 2021; Onodera *et al.*, 2021). According to the classification scheme of Barnes *et al.* (2020), RBD-targeting antibodies can be roughly categorized into four types according to where they bind on the RBD. Class 1 and 2 antibodies bind near the RBD’s ACE2-binding site, and so these tend to be neutralizing antibodies, while class 3 and 4 antibodies bind away from the ACE2-binding site and are less likely to be neutralizing.

Efficient models that can predict RBD binding are incredibly important for the study of viral evolution and vaccine design. Often, the experimental data used to train or validate these models come from deep mutational scanning (DMS), in which RBD variants are randomly generated via yeast surface display (Dong *et al.*, 2021; Francino-Urdaniz *et al.*, 2021; Greaney *et al.*, 2021a, b; Leonard *et al.*, 2022; Starr *et al.*, 2020, 2021a, b; Tyler *et al.*, 2021). Experimental quantities such as ACE2 binding or antibody binding can then be measured for each variant using fluorescence-activated cell sorting.

Previous computational work by Chen *et al.* (2021) focused on the prediction of RBD binding to ACE2 using molecular dynamics (MD) simulations combined with neural networks, which achieved a correlation of 0.73 against binding affinity measurements from DMS. Previous work by us used neural networks to learn RBD-ACE2 binding directly from the DMS data, achieving a correlation of 0.97 without the need for computationally expensive MD simulations (Wang and Chakraborty, 2022). Other methods have been developed to use structures to predict the binding affinity (ΔG) or change in binding affinity upon mutation (ΔΔG) of protein or antigen–antibody complexes (Liu *et al.*, 2021; Moal *et al.*, 2015; Myung *et al.*, 2020, 2022; Pires and Ascher, 2016). Some efforts have been made to develop models that predict protein–protein binding based solely on sequence (Abbasi *et al.*, 2020; Jemimah *et al.*, 2020), but these are comparatively few in number.

In general, most approaches for predicting binding use protein structures, which makes sense because binding is inherently a structural process and contains much more information than the sequence alone. However, the structural requirement limits what data can be used and is also much more computationally expensive because of the work involved in structural optimization and/or MD simulations. The computational cost of prediction is also highly relevant for certain applications. For example, previous models have used structural predictions of antigen–antibody binding affinity to simulate the evolution of antibodies in the secondary lymphoid organs (Conti *et al.*, 2022), a process known as affinity maturation (Victora and Nussenzweig, 2012). For such an application, the prediction needs to be accessible on-the-fly, and it is called thousands of times. Another application is the screening of mutations for protein design, which also requires thousands of predictions. In both cases, long-timescale MD simulations are infeasible and even short-timescale MD simulations and/or structural optimization are costly.

In this work, we have developed a neural network model that predicts antibody binding to SARS-CoV-2 RBDs using the protein sequences and antibody class. This network is trained on DMS measurements of RBD-antibody escape fraction, which is the fraction of cells displaying a particular RBD variant that do not bind to the antibody. Using antibody-RBD measurements that have associated solved structures, we demonstrate that this model yields a higher correlation to DMS measurements than previously published structure-based methods (Jiménez-García *et al.*, 2013; Liu *et al.*, 2021; Mashiach *et al.*, 2008; Myung *et al.*, 2020, 2022; Pires and Ascher, 2016). We also show similar correlation to measurements without associated structures and to dissociation constant measurements against SARS-CoV-2 variants. The residue-level escapes predicted by the model demonstrate that residues in the antibody epitope have the greatest escape, correlating well with escapes measured from DMS. We then study the effects of modifying the network structure by changing the transformer architecture with feed-forward and convolutional architectures, changing the antibody chains used, and changing the number of embedding dimensions. Finally, we show that the inference speed of our model is greater than that of structure-based models, largely because our method does not require the optimization of structure files. We propose the use of our model for applications interested in antibody binding to SARS-CoV-2 RBDs.

## 2 Methods

### 2.1 Dataset and splitting

Measurements of escape fractions from DMS were obtained from the Bloom lab (data found at https://github.com/jbloomlab/SARS2_RBD_Ab_escape_maps) (Cao *et al.*, 2022a, b; Dong *et al.*, 2021; Greaney *et al.*, 2021a, b; Starr *et al.*, 2021a, b; Tortorici *et al.*, 2021; Tyler *et al.*, 2021). In these experiments, the RBD of the Wuhan-Hu-1 strain is taken to be the unmutated RBD, and RBD variants are generated using yeast surface display (multiple cells can display the same RBD). The cells are then sorted into an antibody escape bin based on their lack of binding to a fluorescently tagged antibody. The escape fraction is the fraction of cells displaying a particular RBD that are in the antibody-escape bin. An escape fraction of 0 indicates that no cells displaying a particular RBD are in the antibody-escape bin, and a fraction of 1 indicates that all cells displaying that RBD are in the bin. In the DMS studies, escape fractions are reported for variants that contain a single mutation.

The data also contain the class of each antibody, which range from 1 to 4 and correspond to rough locations on the RBD that the antibodies bind to, as described in Section 1. After collecting the data from multiple studies and removing datapoints for antibodies without reported sequences, the total dataset contains 401 021 datapoints and 1743 different antibodies. Antibody sequences were aligned using ClustalOmega (Sievers *et al.*, 2011) in order to trim sequences containing constant regions such that only variable regions remained. As in the DMS data, RBD residues are defined to range between residue 331 and residue 531.

We created two test sets: ‘WT-Struc’ contains the 4422 datapoints corresponding to two antibodies (C002 and COV2-2196), for which there are experimental structures of these antibodies complexed with the RBD available in the PDB (PDB IDs: 7K8S, 7L7D); ‘WT-NoStruc’ contains 4469 datapoints for 28 antibodies without experimental structures. In total, 4960 datapoints corresponding to 30 antibodies were split into the validation set and 314 773 datapoints corresponding to 1388 antibodies were assigned to the training set. Antibody sequences with >70% sequence similarity threshold based on the CDR3 regions, a value previously used to study antibody–antigen interactions (Sela-Culang *et al.*, 2014), were removed from the dataset. Supplementary Figure S1 illustrates the effect of varying the threshold, demonstrating that the model retains predictive power until a threshold of 50%, at which point the antibodies in the dataset are too dissimilar and too few in number for the model to learn. The two antibodies in the WT-Struc set were not very similar, as the CDR3 sequence similarity was 45%.

We further tested our model against an external dataset from Reincke *et al.* (2022), in which dissociation constants were measured for 11 new antibodies against wild-type, Alpha, Beta, Gamma, Delta and Omicron RBD variants. We refer to this test set as ‘Variants’ to distinguish it from the previous datasets which used the wild-type as a reference.

### 2.2 Network architecture

**Fig. 1.**
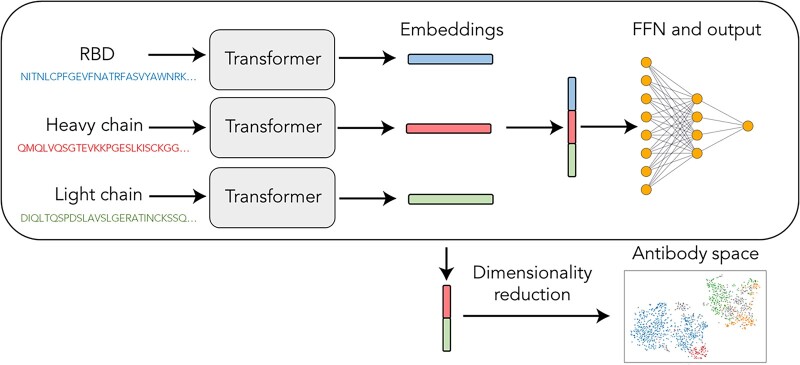
Diagram of the neural network architecture and the construction of the antibody space

The network architecture is illustrated in Figure 1. The heavy chain, light chain and RBD sequence are ordinally encoded and input into an embedding layer to produce input embeddings, which are then processed through a transformer encoder block into a 20-dimensional sequence embedding. The sequence embeddings of the heavy chain, light chain and RBD sequence are concatenated and processed through a feed-forward network. Finally, the output of the network is a scalar—the predicted log escape fraction. The details of the network are provided in the Supplementary Material.

For comparison, we also trained networks using feed-forward (FFN) and convolutional network (CNN) architectures. The details of these networks are also provided in the Supplementary Material.

### 2.3 Residue masking and training

To reduce the network’s dependence on specific sequence contexts, we randomly masked each residue of the antibody sequences during training. Masking a residue means replacing the residue’s amino acid with a mask token, which hides the identity of the amino acid and reduces the amount of information in the sequence. We also masked the residues of the RBD sequence, but certain residues were never masked if they were (1) escape residues for the given antibody class or (2) the mutated residue. Escape residues were residues that produced large mean escapes upon mutation (provided in Supplementary Table S1), and these were previously defined for each antibody class (Greaney *et al.*, 2021a, b). Upon each new epoch, the masked residues were reassigned. The probability of masking each residue for both antibody and RBD sequences was selected using grid search with the validation set, which were found to be 0.75 for both antibody and RBD sequences.

Optimization used Adam, a learning rate of 0.001, a batch size of 256, a mean-squared error loss, up to 200 epochs and early stopping on the validation set with a patience of 10. The model was implemented using PyTorch version 1.9.0 (Paszke *et al.*, 2019).

### 2.4 Antibody sequence clustering

We calculated the heavy and light chain sequence embeddings for every antibody in the dataset and concatenated them (Fig. 1). The concatenated embeddings were then reduced to two dimensions using *t*-distributed stochastic neighbor embedding (t-SNE) with a perplexity of 30. To assign clusters, the pairwise Hamming distance matrix using both heavy and light chains was calculated and then input into a density-based spatial clustering of applications with noise (DBSCAN) algorithm with a Euclidean distance metric, an epsilon of 62 and a minimum number of samples of 35 (Louhichi *et al.*, 2014). t-SNE and DBSCAN algorithms were implemented using scikit-learn version 1.0.2.

### 2.5 Testing and benchmarking

On evaluation, no antibody residues are masked, but RBD residues are still masked according to the rules described above. The prediction is taken to be the mean of 100 outputs, each of which has the residue masks randomly applied to the RBD sequence. In Supplementary Figure S2, we show that averaging over 100 outputs is sufficient to reach convergence. The purpose of masking RBD residues but not antibody residues on evaluation is to differentiate between predictions using different antibody classes, since the antibody class determines which RBD escape residues are never masked.

We calculated Spearman ($\rho_{S}$) and Pearson ($\rho_{P}$) correlation coefficients for our test sets using log-transformed escape fractions as follows:

(1) $$\rho_{S}=\frac{\mathrm{cov}\left(R\left(X\right),\;R\left(Y\right)\right)}{\sigma_{R\left(X\right)}\sigma_{R\left(Y\right)}}$$

(2) $$\rho_{P}=\frac{\mathrm{cov}\left(X,Y\right)}{\sigma_{R\left(X\right)}\sigma_{R\left(Y\right)}},$$

where X is the model prediction, Y is the label, R(X) is the rank variable, σ is the standard deviation and cov is the covariance function.

For comparison, the following existing methods were considered:

1. CSM-AB (Myung *et al.*, 2022): a machine learning method that creates graph-based signatures from structures of antibodies and antigens in order to both score docking poses and calculate binding affinities.
2. PyDock (Jiménez-García *et al.*, 2013): a physics-based scoring function that uses calculations of the electrostatics, implicit desolvation energy and van der Waals interaction in order to perform rigid-body docking.
3. FireDock (Mashiach *et al.*, 2008): a physics-based refinement algorithm and scoring function that focuses on energetic calculations and interfacial side chains to perform rigid-body docking.
4. mCSM-AB (Pires and Ascher, 2016): a machine learning method that uses graph-based signatures from structures of antibody–antigen complexes in order to predict ΔΔG.
5. mCSM-AB2 (Myung *et al.*, 2020): an updated version of mCSM-AB that uses a larger dataset and additional structural and evolutionary signatures in order to predict ΔΔG.
6. GeoPPI (Liu *et al.*, 2021): a machine learning method that predicts ΔΔG that employs self-supervised learning in order to learn an effective geometric representations of the protein structure, which are then used as features for gradient-boosting trees.
7. ISLAND (Abbasi *et al.*, 2020): a machine learning method that predicts ΔG for arbitrary protein sequences using support vector machines.

For the WT-Struc set, we compared our model with all seven models. The models that predict ΔG (CSM-AB, PyDock and FireDock) were calculated using the CSM-AB and CCharPPI webservers (Moal *et al.*, 2015). We selected PyDock and FireDock from the CCharPPI server because these models were the top performers when compared against CSM-AB in Myung *et al.* (2022). The models that predict ΔΔG (mCSM-AB, mCSM-AB2 and GeoPPI) were calculated using the mCSM-AB/mCSM-AB2 webservers and the GeoPPI installation.

For models that predict ΔΔG, we subtracted the predicted log escape fraction of the variant by the log escape fraction of the wild-type RBD sequence. The wild-type escape fraction is not provided in the dataset, so we estimated it as the median escape fraction from non-escape residues for the antibody class. The reasoning is that mutations in these residues are likely to have little impact on antibody binding, and so are representative of wild-type binding. We also assessed the impact of using the 10th percentile instead of the median to account for the phenomenon that most mutations deleteriously affect binding, but this has little impact on the results (Supplementary Table S2).

For the WT-NoStruc set, most of the comparison models were not applicable because the measurements in the WT-NoStruc set do not have associated structures. To assess the performance of our model against a sequence-based predictor, we compared our model with the ISLAND model.

In the Variants set, we removed values for the wild-type RBD to assess the performance of our model strictly on RBD variants, and then calculated correlation coefficients of our predicted log escape fractions to the log dissociation constants. As with the WT-NoStruc set, we calculated values from the ISLAND webserver (Abbasi *et al.*, 2020) for all of our test sets.

We report the Pearson correlation coefficients in the Supplementary Material, but we note that Spearman correlation coefficients are likely more appropriate since the dissociation constants are non-linearly and monotonically related to escape fractions through the Hill equation.

Some previously published models were excluded from testing. The most notable is the SARS-AB model by Beshnova *et al.* (2022), which uses MD simulations to estimate binding energies of SARS-CoV-2 RBDs to neutralizing antibodies. We have excluded SARS-AB because of the high computational cost of performing MD simulations. For our WT-Struc set, which is the only one that has associated structures, there are 4422 datapoints. This is hundreds of times larger than the 20–30 datapoints Beshnova *et al.* (2022) use in their test sets and would require an infeasible amount of computing time. Besides the structural models we test here, there are also many other structural models that predict ΔG or ΔΔG. Rather than performing an exhaustive comparison with all existing models, we have chosen some of the highest performing models from recent years. A sequence-based model for predicting binding affinity that we excluded is ProAffiMuSeq (Jemimah *et al.*, 2020). ProAffiMuSeq requires sufficient homology between the input sequences and sequences within the ProAffiMuSeq dataset. This homology does not exist for our sequences, rendering the method unusable.

## 3 Results

### 3.1 Antibody embeddings encode mutational distances

**Fig. 2.**
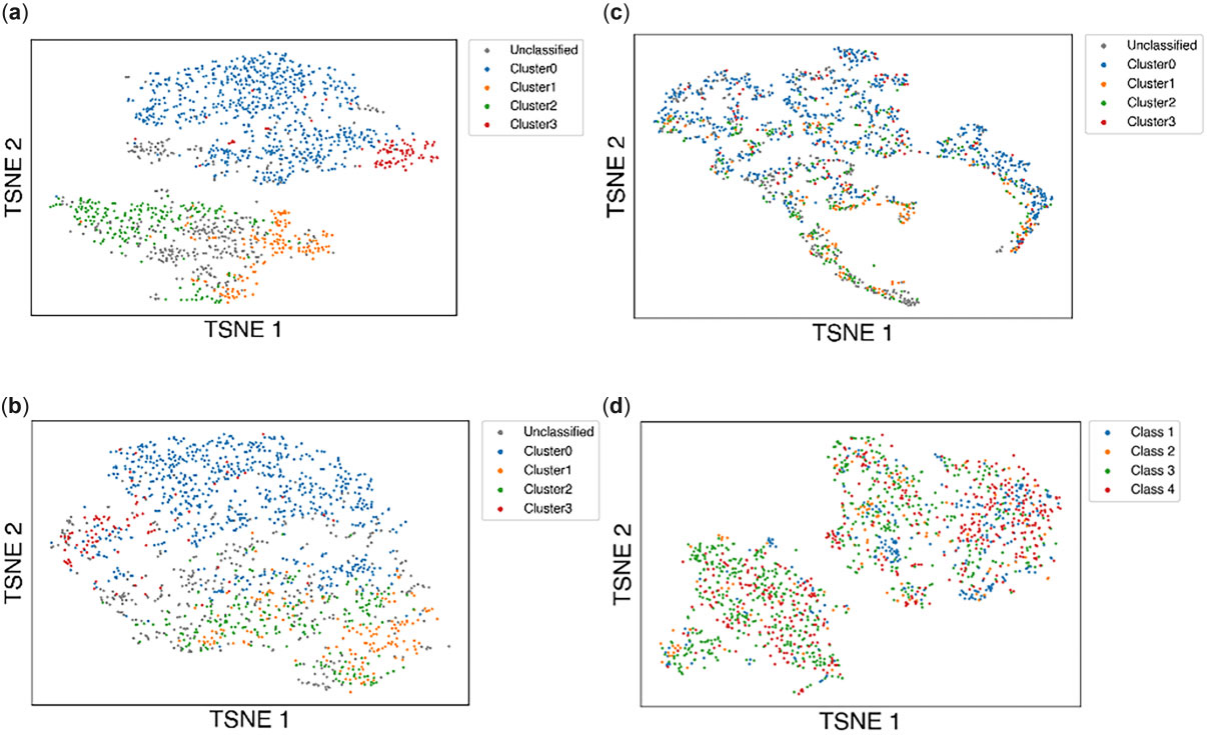
t-SNE plot of antibody embeddings. (**a**) The embedding for each antibody chain is 20 dimensions, and points are colored based on clusters identified through DBSCAN clustering of the Hamming distances between antibody sequences. (**b**) The embedding for each antibody chain is 5 dimensions, and points are colored based on clusters identified from Hamming distances. (**c**) The embedding for each antibody chain is 2 dimensions, and points are colored based on clusters identified from Hamming distances. (**d**) The embedding for each antibody chain is 20 dimensions, and points are colored based on known antibody class

The antibody embeddings are meaningful, in the sense that they constitute a sequence space. After collecting the antibody embeddings for every antibody sequence in our dataset, we show that these embeddings cluster the antibody sequences in a way that strongly correlates to the mutational distances (calculated using the Hamming distance between sequences) (Fig. 2a). Mutational distances (such as the Hamming distance) provide pairwise information but do not assign a particular position in sequence space to a single antibody. On the other hand, an embedding space has the advantage of placing individual antibodies in particular positions in the space while preserving the distances between antibodies. The ability of the antibody embeddings to distinguish different antibodies suggests that they may be useful for downstream tasks such as prediction of binding to RBDs.

Figure 2b and c illustrates the effect of reducing the dimensions of the antibody embeddings. At 5 dimensions, the embeddings are still largely able to cluster antibodies, but not as distinctly as the original model with 20 dimensions. However, at two dimensions, the embeddings are no longer able to cluster the antibodies. The effects of embedding dimension on the model’s ability to predict escape fraction are discussed in Section 3.4.2. Additionally, the 20-dimensional embeddings alone do not cluster antibodies based on the antibody class (Fig. 2d). This highlights the need to provide the antibody class as an input to the model.

Hie *et al.* (2021) demonstrated a similar phenomenon with viral spike sequences. In their case, they directly used the distances between embeddings as a measure of antigenic distance in order to predict viral escape from antibodies. The strategy of constructing embeddings for protein sequences is also commonly found in general protein language models such as Unirep (Alley *et al.*, 2019), ESM (Rives *et al.*, 2021) and TAPE (Rao *et al.*, 2019), which often use these embeddings for downstream tasks such as secondary structure prediction or protein function prediction. Akin to the general protein language models, this work uses these embeddings as input for the downstream task of predicting the escape fraction. However, a difference with our work is that these other models focus on different protein sequences, such as viral spike proteins or all proteins, while this model focuses on antibodies. The focus on antibodies, which have two chains, also leads us to produce two separate embeddings rather than a single embedding. The previous models also generally train their embeddings using unlabeled sequences by providing a self-supervised learning task such as masked residue prediction, whereas this model learns from labeled data. We did explore the possibility of pretraining antibody embeddings using masked residue prediction on unlabeled antibody sequences from the cAb-Rep database (Guo *et al.*, 2019), but using these pretrained embeddings did not improve performance for predicting escape fractions (see Supplementary Table S3, Supplementary Material for details).

### 3.2 Comparison of binding prediction with other methods

**Fig. 3.**
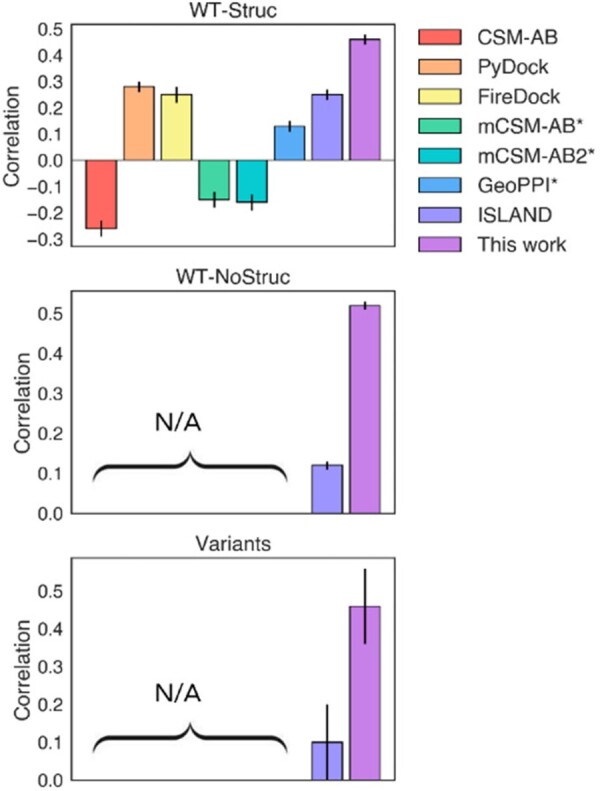
Spearman correlation coefficients of the models against different test sets. * indicates that the model predicts ΔΔ*G* instead of Δ*G*, so for these the log escape fraction of the wild-type RBD was subtracted from the data. Structural models could not be used for the WT-NoStruc and Variants datasets, since these did not have solved structures associated with them. Errors are bootstrapped standard errors

We compared our model against numerous existing structural models and the ISLAND sequence-based model using the WT-Struc set in Figure 3 and Supplementary Table S4. The other models have Spearman correlation coefficients that do not exceed 0.28, and a few such as CSM-AB and mCSM-AB have negative correlations. Our model achieves a 1.6-fold higher correlation of 0.46 and we note similar trends based on the Pearson correlation coefficients (Supplementary Table S5). The previous models were not trained on the SARS-CoV-2-specific data from DMS, but instead on general antibody–antigen binding affinity data, so their performances on a SARS-CoV-2 dataset are likely to be worse than a SARS-CoV-2-specific model. While our model is currently better for SARS-CoV-2, it is possible that training existing models on the SARS-CoV-2 data would improve their performance, subject to the caveat that only some of the datapoints have associated structures. Interestingly, the ISLAND model achieves a similar correlation to several of the structural models despite not having access to structural information. Our model also does not access detailed structural information, but the antibody class is required which does roughly encode which residues are involved in binding.

We further validate our model using the WT-NoStruc set, which contains a larger number of different antibodies. We can compare our model against the ISLAND model because it only uses sequences. However, the complexes in the WT-NoStruc set do not have solved structures associated with them, so comparison with the structural models is not possible. We find that the Spearman correlation coefficient of our model is 0.52, which is relatively similar to the 0.46 correlation from the WT-Struc set. The correlation of the ISLAND model is comparatively lower at 0.12.

The DMS measurements used the Wuhan-Hu-1 RBD sequence as a reference, so the previous tests do not assess the performance of our model on SARS-CoV-2 variants. However, all of the RBD mutations found in SARS-CoV-2 variants are measured in the DMS data, which we hypothesized provides the model with capacity to predict multi-mutant variant binding. Note that since we input the full RBD sequence instead of the mutations, this model does not require averaging over the effect of single mutations. To order to verify our hypothesis, we tested the model using the Variants set. This yields a correlation coefficient of 0.46, which is comparable to correlations from previous tests and is also higher than the ISLAND model’s correlation of 0.10.

### 3.3 Comparison of residue-level escapes with experiment

**Fig. 4.**
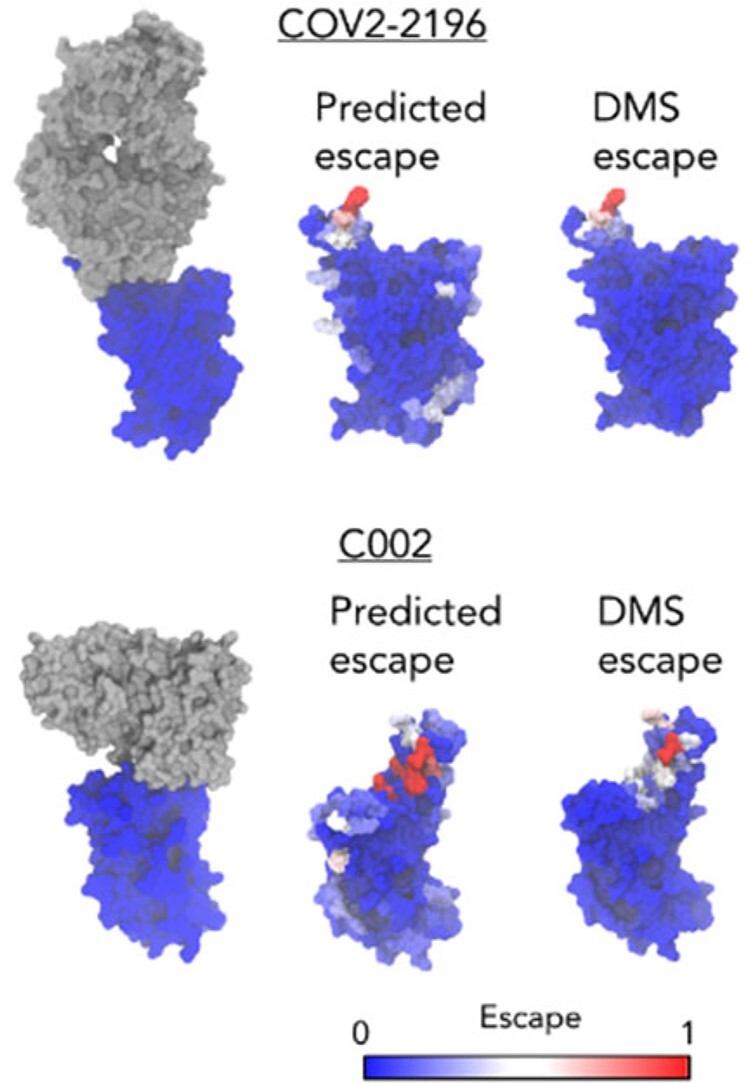
Comparison of predicted residue-level escape with measured escapes from DMS for the COV2-2196 and C002 antibodies. Structures of the antibody–RBD complexes are shown on the left for comparison (RBD: blue, antibody: gray). Complex structures were obtained from the PDB (COV2-2196: 7L7D, C002: 7K8S)

Related to the prediction of escape fractions for individual mutations, we next considered the prediction of escapes for RBD residues. The escape of a residue is defined the same as is done experimentally: the sum of the individual mutational escape fraction at a residue, then normalized across all residues so that a value of 1 corresponds to the larger of the maximal escape at any residue or 20 times the median escape across all residues. In Figure 4, we calculated the escapes of all RBD residues for the two antibodies in the WT-Struc set, and then colored the structure of the RBD based on our predicted escapes and the escapes measured from DMS. One would expect that residues in the antibody epitope would have the greatest escapes. By comparing the DMS escapes and predicted escapes to structures of the antibody–RBD complexes, we see that epitope residues do indeed have the largest escapes. However, the predicted escapes do not perfectly correlate with the DMS escapes. The correlations are 0.53 ± 0.08 for COV2-2196, 0.53 ± 0.1 for C002 and 0.53 ± 0.03 for the WT-NoStruc set. Residues outside of the epitope, sometimes quite distal from the epitope, can have larger predicted escapes. This is because our model does not use structures, so it does not know the proximities of different RBD residues to the antibody. Nonetheless, the ability of the model to identify epitope residues as having the highest escapes is promising.

### 3.4 Effects of network structure

#### 3.4.1 Effect of using a single antibody chain

**Fig. 5.**
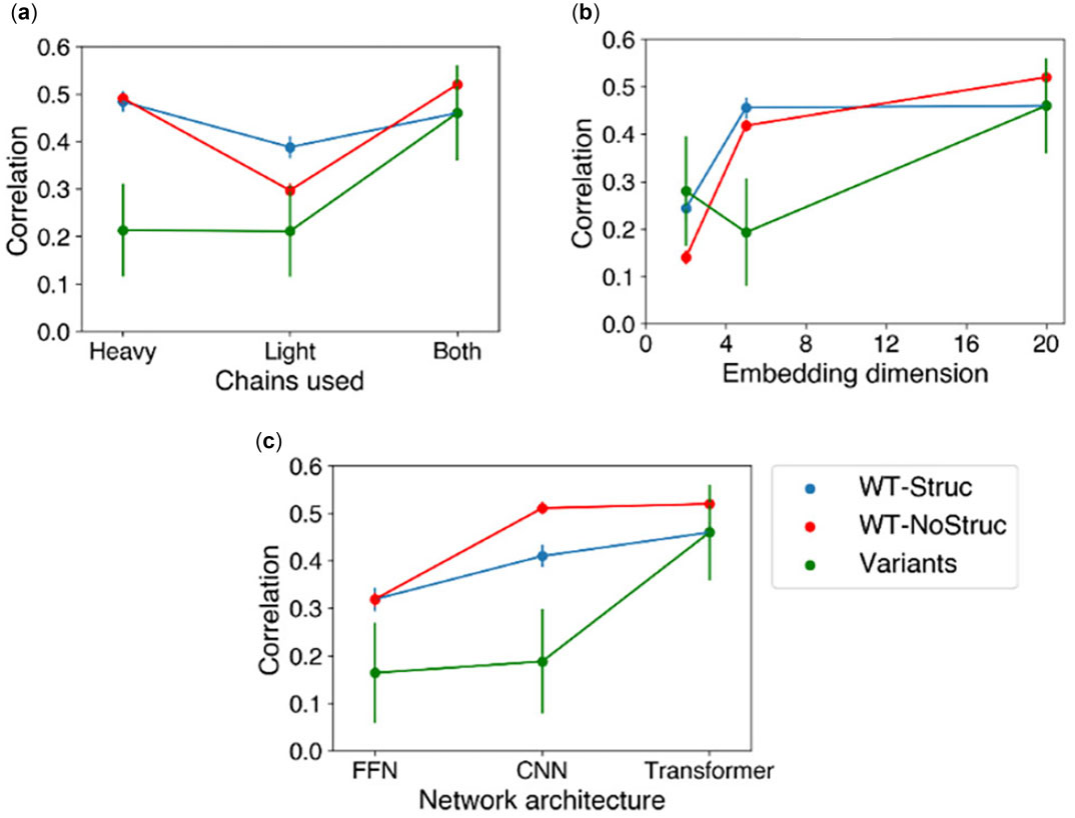
Effects of changes in network structure on test set correlations. (**a**) Spearman correlations for networks using different antibody chains. ‘Both’ corresponds to the original model. (**b**) Spearman correlations for different numbers of dimensions in protein sequence embeddings; 20 corresponds to the original model. (**c**) Spearman correlations for different network architectures. ‘Transformer’ corresponds to the original model

In Figure 5a, the effects of only using the heavy or light chains in the network are illustrated. Based on the WT-Struc and WT-NoStruc sets, the model using only the heavy chain yields higher correlations than those of the model using only the light chain, suggesting that the heavy chain is more important for predicting escape fractions. This is likely due to the greater variance of lengths in the heavy chain compared with the light chain (Supplementary Fig. S3). This variability in the inputs allows the model to distinguish between different antibodies and calculate escape fractions over a larger range (Supplementary Fig. S4). The importance of the heavy chain could also be related to its greater contact area with the antigen and the interactions made by the CDRH3 loop. Although this trend does differ in the Variants set, for which the models using only a single chain perform similarly, this could simply be a product of the greater uncertainty in the correlation coefficient due to the smaller number of datapoints.

#### 3.4.2 Effect of embedding dimension

In Section 3.1, it was illustrated that a small number of dimensions prevents the embeddings from effectively clustering the antibodies. In Figure 5b, we calculated the correlation coefficients against test sets to assess whether the diminished ability to cluster antibodies is associated with a diminished ability to predict escape fractions. Indeed, we observe that the correlation coefficients for the WT-Struc and WT-NoStruc sets track well with the ability of the embeddings to cluster antibodies. The model with an embedding dimension of 5 has slightly reduced correlations compared with the original model with 20 dimensions, while the model with an embedding dimension of 2 has significantly reduced correlations. The models with dimensions of 2 and 5 have reduced correlations on the Variants set but perform similarly to each other, which may be due to the greater uncertainty.

#### 3.4.3 Effect of FFN and CNN architectures

We then studied the effect of using networks based on FFN and CNN architectures rather than a transformer architecture (Fig. 5c). The FFN architecture produces the lowest correlations across all test sets, while the CNN produces correlations between those of the FFN and transformer. This illustrates the benefit of learning a compressed representation of the original sequences, which is present in the CNN and transformer but not the FFN. Ultimately, the transformer provides greater performance compared with the CNN, which is likely due to the inductive biases present in these models. The CNN assumes that local interactions are important. In other words, the CNN assumes that interactions between residues close to each other in sequence are most important. However, interactions between residues far apart in sequence could also be important as such residues may be close in the three-dimensional structure or may interact through allosteric effects. Thus, the inclusion of long-range interactions through the transformer’s attention mechanism could contribute to its greater performance.

### 3.5 Comparison of inference speed with other methods

**Fig. 6.**
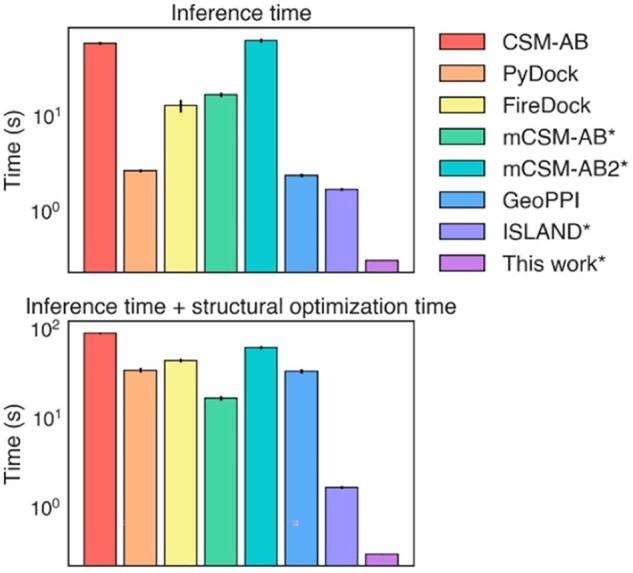
Inference times for a single prediction of each model. The inference time of the model itself is indicated under ‘Inference time’. ‘Inference time + structural optimization time’ includes the time required to optimize structures with a mutated residue. Models that do not require structural optimization or perform it within the model are indicated with an asterisk, and the base inference time is shown for simpler comparison. Errors are standard errors over three independent samples

As previously mentioned, the inference speed of binding prediction methods is incredibly important for certain applications such as simulations of affinity maturation or mutation screening for protein design. We have thus benchmarked the inference times of a single prediction for each model, using either an Intel Core i7-9750H CPU for installed programs or associated webservers, which are reported in Figure 6 and Supplementary Table S6. The structural models have inference times that vary from approximately 2 s to around a minute. Additionally, mutating structures requires additional work because the mutated residue will initially have steric clashes that must be resolved through energy minimization. When this step is included, the inference times of the models become even greater (some models have this step built in, and these are indicated with an asterisk), with no model achieving an inference time below 17 s. By comparison, our model is inferred in 0.306 s, which includes averaging over 100 different randomly masked RBD sequences (the inference time without averaging is 0.006 s). The only other model that exhibits comparable inference times is ISLAND, which is a sequence-based model that also avoids processing structure files.

The time required for structural optimization is a key factor in this comparison. As seen in the GeoPPI and PyDock times, the inference time of the binding model may itself be relatively low, but the time required for prerequisite structural optimization becomes the bottleneck. This particular limitation is inherent to structural models but also largely remedied using sequence-based models.

## 4 Conclusion

The prediction of antibody–antigen binding is a crucial step for applications involving either the physiological or synthetic affinity maturation of antibodies. While many models have been created to predict the binding of antibodies to any antigen, a SARS-CoV-2-specific model may provide increased accuracy for applications specifically involving the SARS-CoV-2 RBD as an antigen. Our model uses a neural network trained on DMS data to predict escape fractions of arbitrary antibody sequences to SARS-CoV-2 RBDs. Our model achieves higher correlations against experimental measurements for wild-type and variant RBDs compared with existing models and has lower inference times. The residue-level escapes also correlate well with experiments and we note the importance of antibody chain choice, embedding dimension size, and network architecture on the model results.

To our knowledge, the SARS-AB model by Beshnova *et al.* (2022), which aims to predict SARS-CoV-2 RBD binding to antibodies, has the most similar goal as ours. However, SARS-AB differs from our model because it requires structures and calculates its predictions using MD simulations rather than directly learning from DMS data. Our model places greater emphasis on fast inference, as it avoids using structure files and MD simulations. The task of Beshnova *et al.* (2022) also differs, as they used their model to classify antibodies as binders or non-binders using known SARS-CoV-2 antibodies versus antibodies randomly chosen from the PDB as their test set, whereas our model aims to quantitatively predict binding. Thus, we believe our model may be more appropriate for applications using quantitative predictions such as protein design.

We believe that our model will be a useful tool for the quantitative prediction of antibody binding to SARS-CoV-2 RBDs. Immediate applications could include the computational design of antibodies or RBD antigens as well as mechanistic simulations of physiological affinity maturation in order to understand adaptive immunity.

## Supplementary Material

vbac103_Supplementary_Data
