# Supplementary material for: Prediction of antibody binding to SARS-CoV-2 RBDs
Source: Bioinform Adv. 2023 Jan 2;3(1):vbac103. doi: 10.1093/bioadv/vbac103 (PMC9868522; doi:10.1093/bioadv/vbac103)
Supplement: vbac103_Supplementary_Data [file vbac103_supplementary_data.docx]

Supplementary Information

Prediction of antibody binding to SARS-CoV-2 RBDs

Eric Wang

Institute for Medical Engineering & Science, Massachusetts Institute of Technology, Cambridge, MA 02139.

Correspondence: [ezw@mit.edu](mailto:ezw@mit.edu)

# Neural network architecture

The protein sequences are ordinally encoded and post-padded with 0s to lengths of 172 for heavy chains, 125 for light chains, and 201 for RBDs. For each protein sequence, token and position embeddings with 36 dimensions are indexed from embedding layers and summed together. The summed embeddings are then input into a transformer encoder block with 6 attention heads, 32 neurons in the dense layers, and ReLU activation. The output of the transformer is averaged across the residue dimension (global average pooling) and input into a Linear layer with 64 neurons and ReLU activation to produce a 20-dimensional output vector. The vectors from each protein chain are concatenated and processed through a FFN containing two layers with 256 and 32 neurons and LeakyReLU activation. The final output is the predicted log escape fraction.

# Feed-forward network architecture

The protein sequences are one-hot encoded with lengths of 172 for heavy chains, 125 for light chains, and 201 for RBDs. The one-hot encoded matrices for each chain are then concatenated in the order of heavy chain, light chain, and RBD. This single matrix is then flattened into a single vector and input into an FFN with 3 layers composed of 1024, 256, and 32 neurons, respectively. Each layer is followed by a ReLU activation function. Lastly, the final output layer is the predicted log escape fraction.

# Convolutional network architecture

The protein sequences are one-hot encoded with lengths of 172 for heavy chains, 125 for light chains, and 201 for RBDs. The one-hot encoded matrices for each chain are then concatenated in the order of heavy chain, light chain, and RBD. The matrix is then fed into a 1D convolutional layer with 100 kernels of size 3, a 1D maxpool layer with kernels of size 3, a 1D convolutional layer with 50 kernels of size 3, and a 1D maxpool layer with kernels of size 3. The output is then flattened into a vector and fed into a Linear layer with 32 neurons. The final output layer is the predicted log escape fraction.

# Neural network with pretrained antibody embeddings

The architecture of this model is nearly identical to that of the network without pretrained embeddings, except that weights in the transformer blocks for the heavy and light chains are pretrained on unlabeled antibody sequences and fixed during training with the escape fraction dataset. We chose not to pretrain RBD embeddings on unlabeled sequences because all RBD mutations are sampled through the DMS procedure.

Unlabeled antibody sequences were obtained from the cAb-Rep database (7194934 heavy chain nucleotide sequences and 1074926 light chain nucleotide sequences). For each dataset, duplicates sequences and sequences containing gaps or ambiguous nucleotides were removed. The nucleotide sequences were then translated into amino acid sequences, and duplicates arising from degenerate codons were removed. 600000 sequences were then randomly sampled for both heavy and light chains. Antibody sequences were ordinally encoded and post-padded to a prescribed length (140 residues for heavy chains and 120 for light chains). The sequences were divided into validation sets containing 3000 sequences, and the rest were used for training.

The tasks of the networks were to predict the amino acid of a masked residue. For each sequence, datapoints were constructed by masking one of the residues, and using the true amino acid as a label. This procedure was done for every residue in the antibody sequence. The architecture of the network was identical to the transformer block in the network without pretrained embeddings, up to the 20-dimensional vector. In this network, the vector is processed into another 20-dimensional output vector. A log-softmax activation is then applied to turn the outputs into log probabilities over the 20 amino acids. A negative log-likelihood loss function was used to calculate the loss with respect to the true amino acids. Training used Adam, a learning rate of 0.001, a batch size of 512, up to 200 epochs, and early stopping on the validation set with a patience of 3.

Embeddings from the heavy chain and light chain networks were then used as inputs predict escape fraction. As in the original network, antibody embeddings were concatenated with the RBD embeddings, and the training procedure was the same.

**Table S1**. RBD escape residues for each antibody class.

| Antibody class | Escape residues |
| --- | --- |
| 1 | 405, 417, 420, 421, 452, 455, 456, 460, 472, 473, 475, 476, 484, 486, 487, 489, 493, 504 |
| 2 | 452, 455, 456, 472, 473, 483, 484, 485, 486, 489, 490, 493, 494 |
| 3 | 346, 439, 440, 443, 444, 445, 446, 447, 448, 449, 450, 452, 490, 494, 496, 498, 499, 500 |
| 4 | 369, 374, 376, 378, 384, 396, 408, 417, 462, 504, 514, 516, 518 |

**Table S2.** Spearman correlation coefficients of models that predict $\Delta\Delta G$ using either the median or 10^th^ percentile escape fraction as the wildtype escape fraction. Correlations are evaluated using the WT-Struc test set. Errors are bootstrapped standard errors.

| Model | Median | 10^th^ percentile |
| --- | --- | --- |
| mCSM-AB | -0.15±0.03 | -0.15±0.03 |
| mCSM-AB2 | 0.16±0.03 | 0.15±0.03 |
| GeoPPI | 0.13±0.02 | 0.13±0.02 |

**Table S3.** Spearman correlation coefficients of the pretrained and original model against different test sets. Errors are bootstrapped standard errors.

| Model | WT-Struc | WT-NoStruc | Variants |
| --- | --- | --- | --- |
| No pretrained embeddings | 0.46±0.02 | 0.52±0.01 | 0.46±0.10 |
| Pretrained antibody embeddings | 0.14±0.03 | 0.18±0.02 | 0.14±0.12 |
|  |  |  |  |

**Table S4.** Spearman correlation coefficients of the models against different test sets. * indicates that the model predicts $\Delta\Delta G$ instead of $\Delta G$, so for these the log escape fraction of the WT RBD was subtracted from the data. Structural models could not be used for the WT-NoStruc and Variants datasets, since these did not have solved structures associated with them. Errors are bootstrapped standard errors.

| Model | WT-Struc | WT-NoStruc | Variants |
| --- | --- | --- | --- |
| CSM-AB | -0.26±0.03 | N/A | N/A |
| PyDock | 0.28±0.02 | N/A | N/A |
| FireDock | 0.25±0.03 | N/A | N/A |
| mCSM-AB* | -0.15±0.03 | N/A | N/A |
| mCSM-AB2* | 0.16±0.03 | N/A | N/A |
| GeoPPI* | 0.13±0.02 | N/A | N/A |
| ISLAND | 0.25±0.02 | 0.12±0.01 | 0.10±0.10 |
| This work | 0.46±0.02 | 0.52±0.01 | 0.46±0.10 |

**Table S5.** Pearson correlation coefficients of the models against different test sets. * indicates that the model predicts $\Delta\Delta G$ instead of $\Delta G$, so for these the log escape fraction of the WT RBD was subtracted from the data. Structural models could not be used for the WT-NoStruc and Variants datasets, since these did not have solved structures associated with them. Errors are bootstrapped standard errors.

| Model | WT-Struc | WT-NoStruc | Variants |
| --- | --- | --- | --- |
| CSM-AB | -0.21±0.04 | N/A | N/A |
| PyDock | 0.41±0.03 | N/A | N/A |
| FireDock | 0.30±0.03 | N/A | N/A |
| mCSM-AB* | -0.19±0.03 | N/A | N/A |
| mCSM-AB2* | 0.19±0.03 | N/A | N/A |
| GeoPPI* | 0.14±0.02 | N/A | N/A |
| ISLAND | 0.32±0.03 | 0.12±0.02 | 0.03±0.09 |
| This work | 0.65±0.02 | 0.49±0.01 | 0.36±0.09 |

**Table S6.** Inference times for a single prediction of each model. The inference time of the model itself is indicated under “Inference time”. “Inference time + structural optimization time” includes the time required to optimize structures with a mutated residue. Models that do not require structural optimization or perform it within the model are indicated with an asterisk, and the base inference time is shown for simpler comparison. Errors are standard errors over 3 independent samples.

| Model | Inference time (s) | Inference time + structural optimization time (s) |
| --- | --- | --- |
| CSM-AB | 59±2 | 91±2 |
| PyDock | 2.7±0.1 | 35±2 |
| FireDock | 13±2 | 45±2 |
| mCSM-AB | 17±1 | 17±1* |
| mCSM-AB2 | 63±3 | 63±3* |
| GeoPPI | 2.4±0.1 | 34±2 |
| ISLAND | 1.71±0.06 | 1.71±0.06* |
| This work | 0.306±0.004 | 0.306±0.004* |


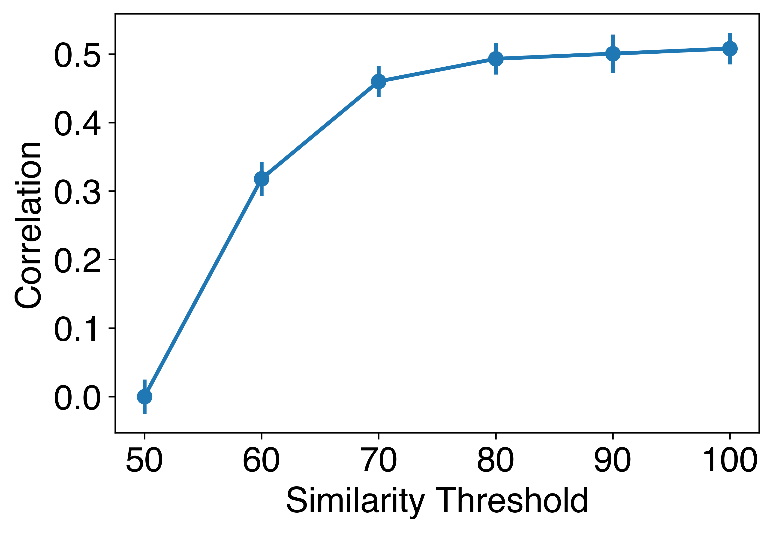


**Figure S1.** Spearman correlation of the WT-Struc test set as a function of the sequence similarity threshold for filtering antibodies.


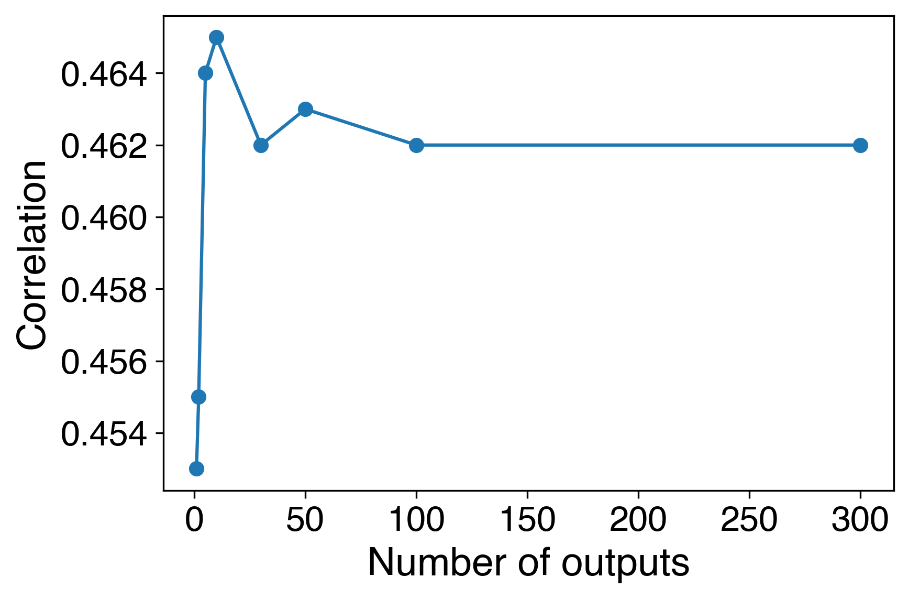


**Figure S2.** Spearman correlation of the WT-Struc test set as a function of the number of outputs averaged over.


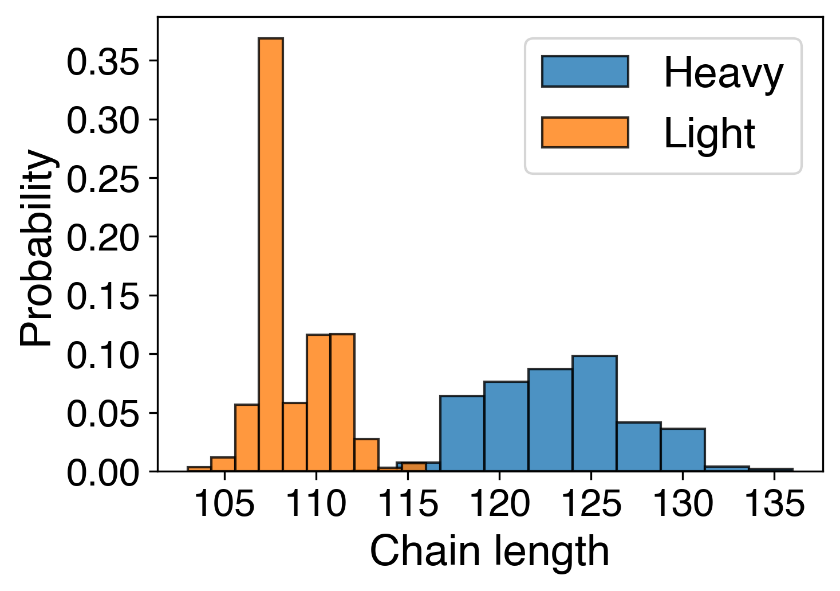


**Figure S3.** Probability distributions of the chain lengths for heavy and light chains in the dataset.


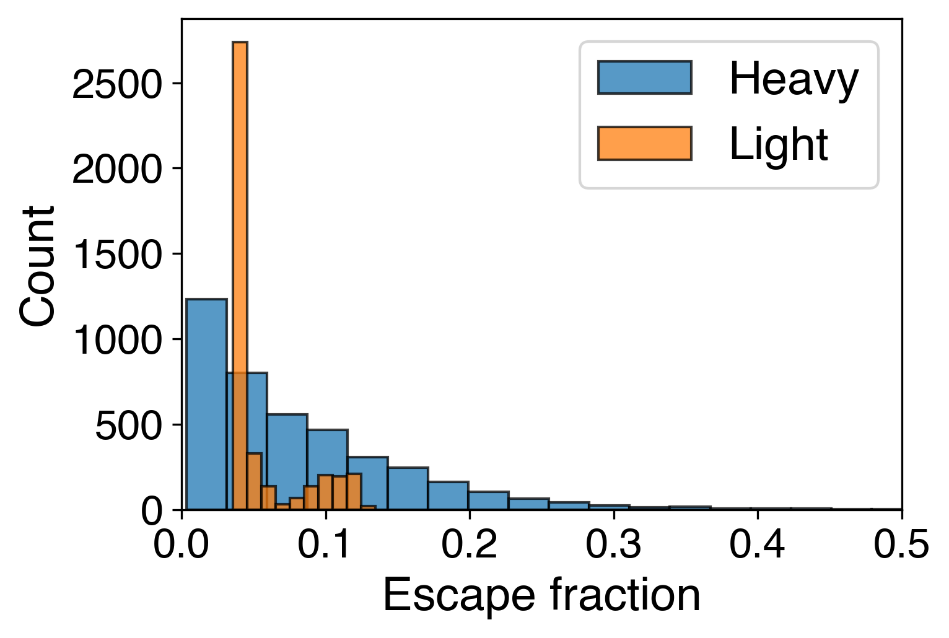


**Figure S4.** Histogram of the escape fractions predicted using either the heavy or light chain for the WT-NoStruc set.
